# Surgical repair of acute on chronic seven‐year pectoralis major rupture near the distal myotendinous junction: A case report

**DOI:** 10.1002/ccr3.6118

**Published:** 2022-07-25

**Authors:** Alvarho J. Guzman, Shane M. Rayos Del Sol, Therese Dela Rueda, Stewart A. Bryant, Sarah Jenkins, Brandon Gardner, Patrick J. McGahan, James L. Chen

**Affiliations:** ^1^ Advanced Orthopaedics and Sports Medicine San Francisco California USA

**Keywords:** bench press, pectoralis major tear, sports medicine, surgical repair

## Abstract

The surgical fixation of an acute on chronic pectoralis major rupture with inciting injury 7 years prior has never been reported in the literature. Thus, we report the first case of an acute on chronic pectoralis major rupture repair in an active male patient who underwent successful surgical intervention and review the pathophysiology and treatment of pectoralis major tears.

## Graphical abstract

Pectoralis major ruptures amenable to surgical repair offer promising outcomes for the patient to return to preinjury level function and athletic performance. We report the first case of surgical fixation for a 7 years acute on chronic rupture.

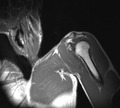

## 1 INTRODUCTION

The pectoralis major (PM) is a thick, fan‐shaped muscle and the most superficial muscle in the pectoral region contributing to thoracobrachial motion. It consists of two heads, the sternocostal and clavicular head, that converge into a single tendon and insert into the crest of the greater tubercle of the humerus. The primary functions of the pectoralis major are to adduct and internally rotate the upper extremity and shoulder joint. Due to its anatomy, the PM is prone to rupture under maximum tension while abducted and externally rotated, with the most common mechanism of injury being the bench press.^1^ Specifically, the inferior portion of the tendon is predisposed to failure first and most reported cases of pectoralis major rupture occur near the distal myotendinous junction due to extreme muscle tension under maximal eccentric overload.^1^, ^2^

Pectoralis major ruptures (PMR), previously thought of as a rare occurrence, have steadily increased in incidence since 1990 with several reviews and large cohort studies detailing this trend.^3^, ^4^, ^5^, ^6^, ^7^, ^8^ Increases in PMR have been attributed to numerous factors including a heightened popularity in health, fitness, weightlifting, and anabolic steroids.^5^, ^7^ PMR can be treated nonoperatively with physical therapy, activity modifications, and oral anti‐inflammatory medications. If a patient fails nonoperative treatment, a PMR can be surgically repaired with an adjustable cortical button or suture anchor fixation.^5^, ^9^ Surgical treatment of PMR is typically recommended and has shown to produce better outcomes in function, strength, patient subjective scores, and return to preinjury performance than nonsurgical management.^5^, ^7^, ^10^, ^11^

To date, there is no literature available describing surgical treatment of an acute on chronic pectoralis major rupture tear with inciting injury 7 years prior. In this case report, we report the surgical repair of a chronic pectoralis major rupture near the distal myotendinous junction in an active male who is expected to make a complete recovery and return to preinjury athletic performance.

## 2 CASE REPORT

A 29‐year‐old right hand dominant male patient presented to our orthopedic clinic with weakness of his left arm abduction. The patient sustained an injury 7 years ago after his left arm was aggressively pulled outwards during a jiu‐jitsu match. He reported experiencing a tearing sensation in his left pectoralis major region when his left arm was in external rotation and abducted 90°. The patient is very physically active and engages in recreational weightlifting, kickboxing, and jiu‐jitsu. On physical examination of the left shoulder and pectoralis, the patient showed full, painless range of motion (ROM) of his left upper extremity (LUE). He demonstrated notable weakness of the LUE in addition to pain with adduction and internal rotation. A positive squeeze test, positive nipple sign, palpable defect, and loss of anterior axillary contour of the left pectoralis major were elicited upon examination. For the past 7 years since the initial injury, the patient reported exhausting several conservative treatment methods with no improvement in his left pectoralis major strength and symptoms. Due to failure of conservative management and a concern for a tear of the left pectoralis major, magnetic resonance imaging (MRI) was ordered to confirm the diagnosis.

**FIGURE 1:**
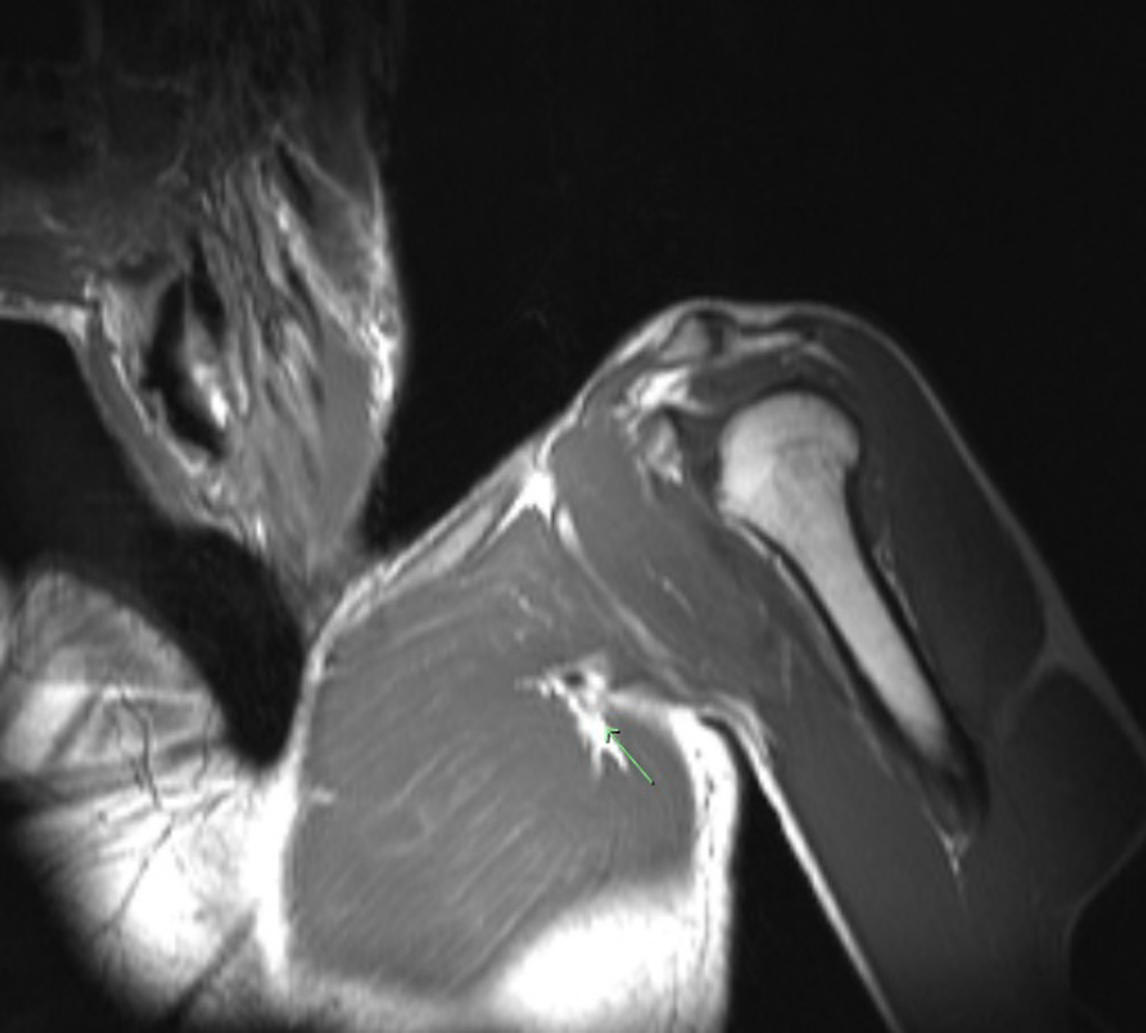
Preoperative T1 coronal imaging of the chest without contrast. A Large high‐grade chronic appearing, likely full‐thickness tear of the sternal head of the left pectoralis major near the distal myotendinous junction is identified by the green arrow

**FIGURE 2:**
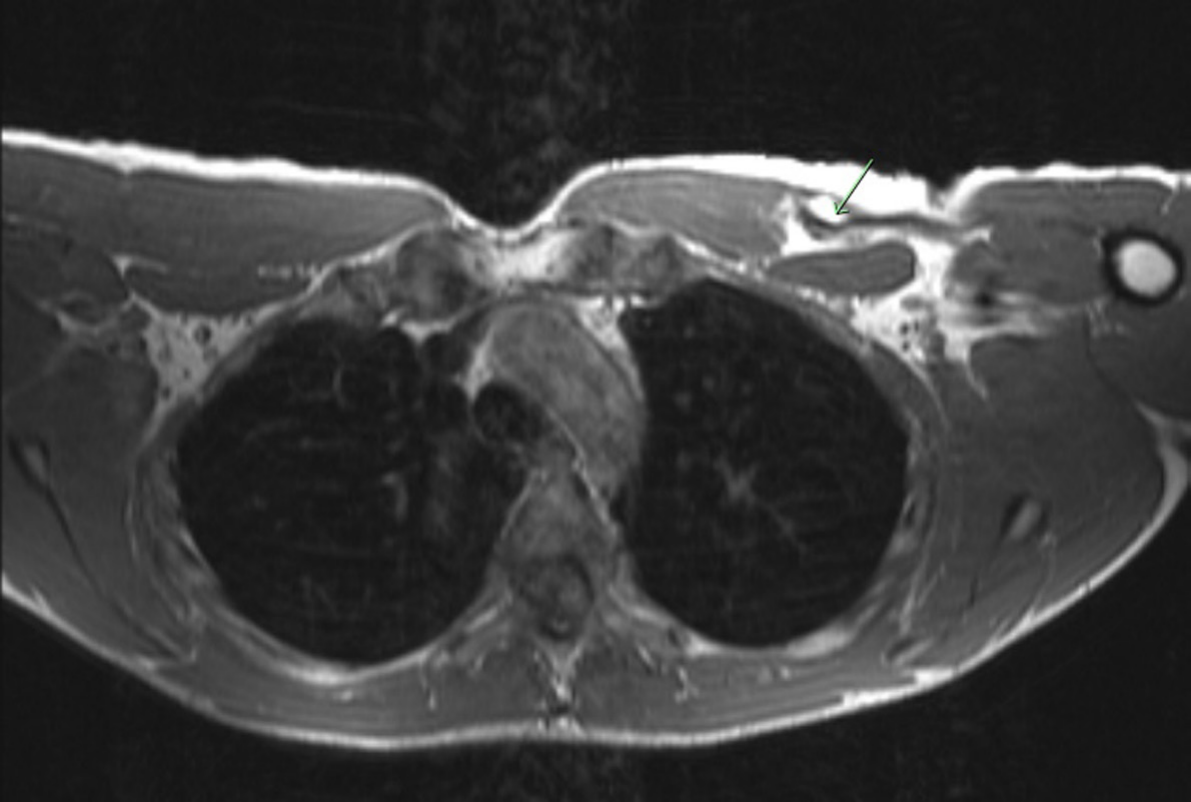
Preoperative T1 axial imaging of the chest without contrast. Full thickness tear of the sternal head of the left pectoral major is again identified by the green arrow

MRI of the chest confirmed a large, high‐grade chronic tear of the sternal head of the left pectoralis major near the distal myotendinous junction (Figures 1 and 2). Furthermore, the MRI was significant for scarring and acute findings of fluid surrounding the chronic tear. Due to chronic weakness, deformity of the left pectoralis, and evidence of acute MRI findings, surgical intervention was discussed with the patient. The risks, benefits, and alternatives to left pectoralis major tendon repair were discussed in detail with the patient and the patient elected to proceed with surgery. The patient was counseled that his expected surgical outcome should include return to sports and weightlifting after 6 months.

The patient was placed in the beach chair position after administration of preoperative antibiotics, a peripheral nerve block, and general anesthesia. After the left upper extremity was prepped and draped in usual sterile fashion, a surgical pen was used to mark the placement of the incision in a longitudinal fashion along the distal deltopectoral groove. With careful dissection, the cephalic vein was protected and retracted laterally and care was taken to identify the retracted, ruptured pectoralis major tendon. The tendon was scarred down, and it was mobilized by freeing the superior and inferior aspects from adhesions of the retracted sternal head with blunt dissection. The insertion site on the anterior humerus lateral to the long head of the biceps tendon was prepared with a Cobb elevator and electrocautery. Once the footprint of the pectoralis major tendon was prepared, the pectoralis major tendon was whipstitched with three FiberTape sutures (Arthrex, Naples FL) along the myotendinous junction and three unicortical drill holes spaced 3 mm apart were created at the native insertion site. Each set of Fibertape sutures was fed through a pectoralis repair button and inserted into the unicortical drill holes, returning the tendon back to its native footprint.

Postoperatively, the patient was placed in a sling for 6 weeks. He was instructed to be non‐weight bearing, perform passive shoulder forward flexion and pendulum exercises only, and avoid shoulder abduction or external rotation. At his first follow‐up visit 2 weeks after surgery, he presented with a moderate‐sized hematoma over the medial biceps near the left pectoralis major insertion. Ninety milliliters of bloody fluid was aspirated in office without complications; then, the patient began physical therapy per pectoralis major repair protocol. At his 8 weeks follow‐up, the hematoma had not reformed and the patient was doing well. At 4 months postoperatively, his hematoma had resolved on its own. There was some discoloration over the left anterior shoulder and pec insertion from cupping administered during physical therapy that was visible (Video S1). The patient had started strengthening in physical therapy and was able to do pushups at parallel with minimal pain. At 6 months postoperatively, the patient was bench pressing 145 pounds, nearly 70% of his one rep maximum prior to the injury sustained 7 years ago. He reported experiencing no weakness or limitation in ROM of his LUE. At this point, the patient was cleared for full activity under the guidance of a physical therapist at 6 months postoperatively (Video S2). He is expected to make a full recovery and return to preinjury level performance in the next few months.

The authors obtained the patient's informed consent for print and electronic publication of the case report before submission.

## 3 DISCUSSION

Classically a rare injury, pectoralis major ruptures have been steadily increasing in frequency largely due to a growing trend and emphasis on more active, physically demanding lifestyles and training regimens.^3^, ^4^, ^5^, ^6^, ^7^, ^8^ PMR are commonly reported in active males aged 20–40, with the greatest incidence of ruptures occurring from eccentric overload during the bench press exercise.^3^, ^4^, ^5^, ^6^, ^7^, ^8^, ^12^ However, a 2012 systematic review by Elmarghy et al. identified 11 cases of PMR in elderly women aged 73 to 97.^8^ Additionally, two case reports of PMR in active females have been reported that were both caused by dynamic upper body trauma. These patients underwent surgical fixation afterward and resumed full functional activity postoperatively.^13^, ^14^ The occurrence of PMR in differing patient demographic categories thus highlights the increasing incidence of injury in diverse patient groups.^3^, ^10^, ^15^, ^16^, ^17^ Chronic pectoralis major ruptures amenable to surgical fixation have rarely been reported in medical literature.^3^ Chronic pectoralis ruptures are often difficult to treat due to shortening of the tendon that may prohibit secure fixation.^13^ Therefore, we present the first reported surgical repair of an acute on chronic 7 years pectoralis major rupture.

Return to sport outcomes following pectoralis major rupture repair are very promising in the current literature. A 2019 systematic review by Yu et al. of 2332 articles and 536 patients who underwent pectoralis major rupture repair found a 90% return to sport after a mean of 6.1 months postoperatively, with nearly 65% of patients consisting of amateur athletes.^11^ Moreover, 75% of these patients returned to preinjury level of athletic performance.^11^ The return to sport rate is similar in competitive and professional athletes, but the more elite athlete returns to preinjury level athletic performance at suboptimal performance level.^18^ For instance, a study by Guity et al. of 24 professional bodybuilders revealed that 21 of these athletes returned to sport but only 6 athletes returned to preinjury level sport in addition to experiencing a nearly 10% strength loss relative to the contralateral arm.^18^ Furthermore, a 2020 study of NFL athletes spanning 15 consecutive years by Sahota et al. demonstrated that players with PMR treated surgically missed significantly more time than those treated nonoperatively, but there was no significant difference in mean games missed after athletes were treated operatively for PMR and nonoperatively for pectoralis major strains.^19^ However, pectoralis major strains versus PMR are injuries of different severities, which may explain the lack of difference reported. All in all, the operative management of pectoralis major ruptures shows promising functional outcomes in the amateur and elite athlete to return to sport and preinjury athletic capacity.

In comparison with nonoperative management of pectoralis major ruptures, surgical intervention has proven to be superior in regard to improving strength, function, satisfaction, and cosmesis.^5^, ^6^, ^7^, ^10^, ^11^, ^15^ A systematic review by Bak et al. highlights the stark contrast between surgical and nonoperative treatment of PMR, revealing that out of 112 cases, 88% of patients experienced good to excellent clinical results with surgical repair.^20^ Moreover, studies have shown that surgically treated individuals display greater recovery of peak torque, restoration of normal strength, and work performance/repetition compared to conservative management of patients with rupture of the pectoralis major.^21^, ^22^ A prospective randomized clinical trial by Pochini et al. further demonstrated the benefit of surgical repair for PMR and found that operative treatment resulted in excellent outcomes at 4 years follow‐up.^6^ This same study demonstrated the bench press was associated with 80% of PMR, excellent outcomes were not reported in any patients treated nonoperatively, and a significant 41.7% decrease in strength measured by isokinetic evaluation of the nonsurgical group relative to 14.3% in the surgical group.^6^

Acute surgical repair for PMR is typically recommended, given that early surgical fixation can avoid the development of chronic lesions and retraction of the ruptured PM.^3^, ^7^, ^23^ Studies have shown that surgery within 8 weeks demonstrates better functional outcomes than delayed surgery, and delayed surgery demonstrates better functional outcomes than nonoperative management.^3^, ^20^ Although several studies report slightly improved functional outcomes for acute repairs of PMR in comparison with chronic repair, all these studies lacked statistical significance and failed to demonstrate a notable difference between acute and chronic surgical groups.^22^, ^23^, ^24^ Additionally, excellent results following repair of chronic PMR are highly substantiated in available medical literature.^1^, ^3^, ^16^, ^23^, ^24^, ^25^ Surgical repair for chronic ruptures of other muscles has also been well reported with good reported functional recovery.^26^, ^27^, ^28^ Given our patient's chronic weakness of the affected arm, MRI findings of acute injury 7 years after injury, and the patient's wish to continue an active lifestyle, surgical intervention was recommended to the patient. At 6 months postoperatively, our patient demonstrated no weakness of the LUE and was bench pressing 70% of his one rep maximum before his initial injury 7 years ago and surgery. Our case report highlights the surgical repair of an acute on chronic 7 years PMR in an active male who is expected to make a full recovery and return to preinjury level athletic performance in the next 3 months, further exhibiting the efficacy of surgical treatment for acute on chronic muscular ruptures.

## 4 CONCLUSION

This case report highlights the surgical repair of an acute on chronic pectoralis major rupture with inciting injury 7 years prior that provided reliable shoulder strength and function, allowing a return to preinjury athletic ability and functional activity. We wish to emphasize the feasibility and efficacy of surgically fixation of an acute on chronic muscular ruptures in an active, motivated patient. Surgical repair may allow for complete functional recovery and restoration of full strength of the muscle, as evident in this patient's case. With this case report, we advocate for surgical fixation as a suitable and effective option to treat acute on chronic pectoralis major ruptures.

## AUTHOR CONTRIBUTIONS

Alvarho Guzman, BA involved in substantial contributions to the conception or design of the work; or the acquisition, analysis, or interpretation of data for the work. Drafting the work or revising it critically for important intellectual content. Agreement to be accountable for all aspects of the work in ensuring that questions related to the accuracy or integrity of any part of the work are appropriately investigated and resolved. Shane M. Rayos Del Sol, MS involved in substantial contributions to the conception or design of the work; or the acquisition, analysis, or interpretation of data for the work. Drafting the work or revising it critically for important intellectual content. Therese Dela Rueda, BS, Stewart A. Bryant, MD, and Sarah Jenkins, MD involved in drafting the work or revising it critically for important intellectual content. Brandon Gardner, MD, PhD involved in drafting the work or revising it critically for important intellectual content. Final approval of the version to be published. Patrick J. McGahan, MD and James L. Chen, MD, MPH involved in final approval of the version to be published.

## CONFLICT OF INTEREST

J.L.C. is an educational consultant for Arthrex and receives compensation for medical educational lectures and instruction only.

## CONSENT

Written informed consent was obtained from the patient to publish this report in accordance with the journal's patient consent policy.

## Supporting information


Video S1

Video S2

Data S1

## Data Availability

Data sharing is not applicable to this article as no new data were created or analyzed in this study.
