# Supplementary material for: Surgical repair of acute on chronic seven‐year pectoralis major rupture near the distal myotendinous junction: A case report
Source: Clin Case Rep. 2022 Jul 25;10(7):e6118. doi: 10.1002/ccr3.6118 (PMC9309739; doi:10.1002/ccr3.6118)
Supplement: Supplementary file 3 — Data S1 [file CCR3-10-e6118-s001.docx]

Video S1 In office video at the 4 months postoperative period. Normal anterior axillary contour of the left pectoralis major is appreciated on physical examination. Discoloration over the left anterior shoulder and pec insertion are visible from cupping administered during physical therapy. The hematoma which had appeared 2 weeks following surgery and was subsequently aspirated had not resumed and resolved on its own after drainage.

Video S2 In office video at the 6 months postoperative period. Normal anterior axillary contour is noted in addition to good strength of the left pectoralis major on physical examination. Discoloration from cupping therapy is visible over the left anterior shoulder and pec insertion. The patient was cleared to full activity following this visit and advised to return on an as needed basis.
